# The Role of Gut Microbiota and Its Metabolites in Mitigating Radiation Damage

**DOI:** 10.3390/microorganisms13092151

**Published:** 2025-09-15

**Authors:** Hansheng Zhu, Xin Yan, Hao Shi, Yiping Chen, Changyi Huang, Yue Zhou, Shiying Yan, Nan Zhang, Jia Wang, Jian Zhang, Chaoyi Han, Qian Chen, Jian Zhao, Mei Cao

**Affiliations:** 1 Key Laboratory of Biological Resource and Ecological Environment of Chinese Education Ministry, College of Life Sciences, Sichuan University, Chengdu 610064, China; zhuhansheng_0405@163.com (H.Z.); 13678146781@163.com (X.Y.); sh744426@stu.scu.edu.cn (H.S.); chenyiping@stu.scu.edu.cn (Y.C.); huangchangyi@stu.scu.edu.cn (C.H.); zhouyue08@stu.scu.edu.cn (Y.Z.); 13107178792@163.com (S.Y.); 2022141450336@stu.scu.edu.cn (N.Z.); gretaguitar@foxmail.com (J.W.); 13644919117@163.com (J.Z.); 2 Core Laboratory, School of Medicine, Sichuan Provincial People’s Hospital Affiliated to University of Electronic Science and Technology of China, Chengdu 610072, China; hanchaoyi0114@163.com; 3 Irradiation Preservation and Effect Key Laboratory of Sichuan Province, Sichuan Institute of Atomic Energy, Chengdu 610101, China; ggcqlz@163.com

**Keywords:** ionizing radiation, intestinal dysbiosis, intestinal microbiota metabolites, radiation-induced intestinal damage, irradiation protection measures

## Abstract

With the widespread use of ionizing radiation (IR) in medical and industrial settings, irradiation has become increasingly common, posing significant risks to human health. Among the various organs affected, the gut is particularly sensitive to radiation-induced damage, leading to conditions such as radiation-induced intestinal damage (RIID). Recent studies have emphasized the critical role of gut microbiota and its metabolites in mitigating radiation-induced injury. This review discusses the effects of IR on the mammalian and human gut microbiota. We examine the dynamics of gut microbiota composition during and after irradiation, and emphasize the protective role of the gut flora and the metabolites in the pathophysiological mechanisms exhibited during radiation injury. In addition, this article investigates how specific metabolites, such as short-chain fatty acids and indole derivatives, may contribute to the mitigation of inflammation and promotion of gut barrier integrity. In addition, various therapeutic strategies based on modulating the gut microbiota, such as probiotics, antibiotics, and fecal microbiota transplantation, are discussed to understand their potential to prevent or mitigate RIID. Understanding the interactions between IR, gut microbiota and their metabolites provides new avenues for developing innovative therapeutic approaches to improve patient outcomes during and after radiotherapy. Future research directions could focus on optimizing microbiota-based therapies and exploring the role of diet and lifestyle in enhancing intestinal health during irradiation.

## 1. Introduction

With the widespread use of ionizing radiation (IR) in research, industry, homeland security, and modern medicine, it has become virtually unavoidable for humans to be exposed to low doses of artificial IR on a daily basis [1]. IR is increasingly used in medicine, which is one of the largest sources of IR exposure as a diagnostic and therapeutic modality [2]. Most hospitals around the world utilize radiation for medical diagnosis and radiotherapy. Although this technology has brought benefits to the treatment of human diseases, it can also cause some damage to living organisms and side effects, which can lead to the serious ionization of biological macromolecules or chromosomal aberrations and mutations [3,4,5]. Different organs have different sensitivities to radiation and therefore the biological effects of radiation on different organs vary [6]. The gut tract, particularly gut epithelial cells and crypt stem cells, is radiation-sensitive and the biological effects as well as protection from radiation have been extensively investigated. Radiation-induced intestinal damage (RIID) is one of the most common diseases following radiation exposure [7].

The gut microbiota is inseparably associated with gut health. The gut microbiota forms a symbiotic association with the host, mainly through its metabolites, maintaining a dynamic balance between the pathological and physiological processes of the host. But any alteration to this balance leads to ecological disorders [8,9]. Studies have shown dynamic changes in the composition of the gut microbiota during and after radiotherapy for disease [10]. Irradiation therapy leads to an increase/decrease in the abundance of some specific gut microorganisms, which may induce abnormal conditions/health states [11]. Gastrointestinal (GI) complications, such as inflammatory bowel disease (IBD), have also been demonstrated to be correlated with radiation-induced disruption of the gut microbial homeostasis [12,13], whereas some microbiota will provide protection to the host from high doses of radiation by promoting hematopoietic proliferation and reducing cell death.

A number of technological and biological strategies have been developed to prevent and mitigate radiation damage to the gut, such as prophylactic surgical techniques, endoscopic treatments, topical treatments, anti-inflammatory drugs, and biological, chemical, and pharmacological therapies [14]. However, these methods also have certain limitations and risks [15]. In recent years, the relationship between radiation and the gut microbiota, as well as the relationship between the gut microbiota, its metabolites and RIID, has attracted the attention of researchers. The treatment of radiation enteritis has progressively focused on remodeling the microbiota or restoring its integrity [16,17]. Microbiota modulation may be an effective way to reduce radiation-induced GI syndromes [18].

Therefore, elucidating the link between IR, the gut microbiota and its metabolites is essential for the development of radiation injury-related preventive therapies. In this review, we will discuss the effects of IR on the gut microbiota of mammals and humans and the link between the gut microbiota and ionizing radiation-induced injuries. We hope to emphasize the protective role of the gut flora and their metabolites in the pathophysiological mechanisms manifested during radiation injury. The advantages and efficacy of modulating the gut microbiota are also discussed, and relevant therapies based on the gut flora and its metabolites are outlined. Finally, we outline the future directions of this approach for the prevention and treatment of radiation-induced injury. This study aims to clarify the potential of the gut flora and its metabolites and to explore new avenues of research and preventive therapeutic strategies for GI injuries induced by radiation exposure.

## 2. The Role of the Gut Microbiota in Human Health

The human gut is a remarkably complex and diverse microecosystem, home to trillions of microorganisms that play essential roles in maintaining host health [19,20]. This intricate ecosystem is composed of various microbes, including bacteria, viruses, fungi, and archaea [21].

The gut microbiota plays an important role in material metabolism, biological barriers, immune regulation and host defense [22,23,24,25]. The microbes in the GI tract assist in breaking down complex carbohydrates, fats, bile salts, and fat-soluble vitamins. They also utilize a diverse array of enzymes that the human body alone cannot produce. This microbial activity not only facilitates the efficient extraction of energy from food but also contributes to the synthesis of essential nutrients, such as short-chain fatty acids, which play a vital role in maintaining intestinal health [21,26]. In addition to their metabolic functions, gut microbes are deeply involved in regulating multiple metabolic pathways within the host. They produce metabolites that can influence distant organs, establishing a physiological link between the gut and other key systems, including the liver, muscles, and brain [27]. This gut–organ axis underscores the significance of the microbiota in overall health, as alterations in microbial composition can have far-reaching consequences. For instance, changes in gut microbiota can modulate immune responses, enhancing or impairing the body’s ability to fight off infections and other diseases.

Recent studies have highlighted the profound impact of gut microbiota on host health, particularly in relation to dysbiosis—a state of microbial imbalance [28]. Dysbiosis has been associated with a wide range of diseases, including metabolic disorders, inflammatory conditions, and even certain cancers [29,30]. The disruption of the normal microbial community can lead to altered intestinal metabolites, which may directly damage the intestinal lining or indirectly impair gut function by disrupting communication between the gut and other organs. In the context of colorectal cancer, research has shown that germ-free animal models with antibiotic-induced dysbiosis (GF-AOMS) exhibited an increased abundance of *Enterobacteriaceae* and secondary bile acids like taurodeoxycholic acid (TDCA). These changes were linked to the activation of the MAPK/ERK pathway and impairment of the intestinal barrier, ultimately compromising the integrity of the colonic epithelium [31].

Moreover, the microbial transformation of certain substances has emerged as a key metabolic marker in the progression of various diseases [32]. As research into the gut microbiota and its metabolites continues to deepen, there is growing interest in the potential of manipulating the microbial community to prevent or treat disease [27,33,34]. By targeting specific microbes or modulating the production of particular metabolites, it may be possible to restore balance within the gut ecosystem and improve host health outcomes. In summary, the gut microbiota is an indispensable component of human physiology, with its influence extending far beyond the GI tract. Understanding the dynamics of this microbial ecosystem and its interactions with the host is crucial for developing innovative strategies to prevent and treat diseases associated with dysbiosis. As scientific knowledge in this field expands, the potential for microbiota-based therapies continues to offer promising avenues for improving health and combating a range of conditions.

## 3. Radiation-Induced Intestinal Damage

The gut is both a highly radiosensitive and an important dose-limiting organ. So radiation therapy for tumors of the abdominal, pelvic, and colorectal systems will typically develop RIID, leading to side effects that include vomiting, weight loss, anorexia, dehydration, diarrhea, and infections [35,36,37,38]. RIID is categorized as either acute or chronic. Acute RIID is mainly characterized by crypt cell apoptosis, which leads to usually reversible rupture of the epithelial barrier and subsequent inflammation [39]. Chronic RIID is delayed, which leads to loss of GI function and is associated with vascular sclerosis and fibrosis of the GI wall [40,41,42].

### 3.1. Cellular Damage and Inflammation

IR causes damage at the cellular level mainly by inducing DNA breaks, leading to cell death or dysfunction [39]. The intestinal epithelium consists of rapidly dividing cells that line the intestines and provide an important barrier against harmful substances entering the body [43]. Due to the high proliferation rate of intestinal epithelial cells, it is particularly vulnerable to radiation, and within the intestinal crypt stem cells, there exists a “hypersensitive subpopulation” that is more vulnerable to damage than proliferating cells or even lymphocytes [40,44]. When exposed to IR, the integrity of the intestinal barrier is compromised, leading to an increase in intestinal permeability, which can make it easier for pathogens and toxins to pass through the intestinal wall and trigger an inflammatory response [45,46].

The inflammatory response induced by radiation injury leads to the upregulation of various pro-inflammatory cytokines, including tumor necrosis factor-alpha (TNF-alpha), interleukin-6 (IL-6), and interleukin-1β (IL-1β), which play a key role in amplifying the inflammatory response and recruiting immune cells to the site of injury [40,47,48,49]. However, this response, while part of the body’s attempt to repair tissue, can exacerbate damage if it becomes chronic or excessive, leading to tissue inflammation and disruption of the mucosal barrier [50,51]. The persistence of these inflammatory mediators not only leads to further epithelial cell death and dysfunction, it also leads to disruption of the extracellular matrix and intercellular tight junctions, thus exacerbating barrier dysfunction [52].

### 3.2. Vascular Injury with Hypoxia and Fibrosis

IR severely damages the microvascular system within the intestinal tissues, injuring endothelial cells and destroying the integrity of the vascular barrier, leading to increased vascular permeability and reduced blood flow [53]. This leads to a hypoxic environment where tissues are deprived of essential oxygen and nutrients. This hypoxia not only exacerbates cellular stress, but also triggers a pro-inflammatory response [54]. The inflammatory environment recruits a variety of immune cells to the site of injury, releasing cytokines and other mediators that further propagate tissue damage, which further leads to increased vascular permeability and vasodilation [55,56]. Over time, ongoing inflammation leads to fibrosis, which can cause the intestinal wall to harden, disrupting its normal function and leading to long-term complications. The interplay between endothelial cell damage, decreased blood perfusion, hypoxia, and subsequent inflammation and fibrosis creates a vicious cycle that ultimately exacerbates damage to the intestinal wall following radiation exposure [40,57,58].

### 3.3. Chronic Radiation-Induced Intestinal Damage

While most epithelial and microvascular injuries occur during the acute phase of irradiation, unresolved epithelial barrier defects coupled with progressive vascular injury and fibrosis can evolve into chronic radiation-induced intestinal damage (RIID). Clinically, chronic RIID typically manifests after a latency of ≥90 days—often months to years—following radiotherapy, presenting with persistent diarrhea, malabsorption, abdominal pain, rectal bleeding, strictures, fistulae, and even obstruction, with substantial detriments to quality of life [59,60]. Notably, ~90% of patients experience permanent changes in bowel habits, and 30–66% of pelvic cancer survivors report chronic gastrointestinal symptoms, underscoring the magnitude of the problem [40,61,62].

Pathobiologically, chronic RIID is characterized by sustained endothelial dysfunction, vascular sclerosis, tissue hypoperfusion/hypoxia, and progressive fibrosis, culminating in mural thickening and mechanical stiffness [63,64]. Repetitive or fractionated irradiation reduces mucosal perfusion and oxygen delivery, perpetuates oxidative stress, and activates fibroblasts to drive excessive extracellular-matrix deposition and collagen remodeling; these events are reinforced by long-term immune-cell infiltration and persistent up-regulation of TNF-α, IL-6, and profibrotic mediators such as TGF-β, creating a self-amplifying loop that differentiates chronic from (potentially reversible) acute injury [65,66,67].

The natural history of chronic RIID reflects this multicellular cascade: (i) vascular rarefaction and barrier failure sustain ischemia and low-grade inflammation [65]; (ii) persistent myofibroblasts and extracellular matrix remodeling consolidate strictures and stiffness [68]; and (iii) attrition of the intestinal stem-cell niche limits crypt regeneration, predisposing to epithelial atrophy and ulceration [69]. Because multiple bowel segments can lie within the radiation field, symptom clusters often arise from more than a single physiological driver and may progress despite apparent resolution of early acute toxicity.

## 4. Interaction Between Gut Microbiota, Its Metabolites and Radiological Bowel Injury

### 4.1. Changes in Gut Microbiota and Its Metabolites After Radiotherapy

In a healthy state, the composition and species of the gut microbiota remain in a relatively stable state, and the main phyla of the gut microbiota are *Firmicutes*, *Bacteroidetes*, *Proteobacteria*, *Actinobacteria*, and *Fusobacteria*, of which the proportion of *Firmicutes* and *Bacteroidetes* is largest [70,71]. IR causes significant changes in the gut flora of organisms, and high doses of radiation directly kill microbial cells in the gut (Table 1), thereby reducing overall microbial diversity and abundance [7,72,73].

Analysis of changes in gut microbial communities based on high-throughput sequencing techniques (marker gene sequencing and whole-genome shotgun metagenomics) revealed that changes in gut flora and their metabolites were not constant after radiation, which may be related to radiation duration, radiation level, radiation type and experimental subjects [52,80,81]. The following section focuses on the effects of IR on the intestinal flora of mammals and humans (Table 2).

As can be seen from Table 1, the ratio of *Bacteroidetes* to *Firmicutes* decreased in most cases, while the number of *Helicobacter* and *Clostridium* increased, while the metabolites that showed significant differences were mainly related to short-chain fatty acids, lipids and amino acids [12,72,81,82,83,84,85]. And we found that most of the previous studies have focused on higher doses of acute radiation therapy, probably due to equipment limitations and other reasons. However, radiation therapy is a longer cycle, and there are few studies on low-dose, long-term radiation therapy, which is a weak point in the field of radiation research and deserves more in-depth studies in the future.

### 4.2. The Role of Gut Microbiota and Its Metabolites in Radiation-Induced Intestinal Damage

The interactions between IR, host response and the gut microbiota are complex, and the exact underlying mechanisms are still under investigation [86]. Intestinal flora and their metabolites may attenuate radiation-induced injury and reduce pro-inflammatory responses to prevent or minimize radiation-induced injury, such as short-chain fatty acids, indole derivatives, and other intestinal microbiota metabolites, and have long-term radioprotective effects [13,82].

#### 4.2.1. Short-Chain Fatty Acids (SCFAs)

SCFAs such as acetate (C2), propionate (C3), and butyrate (C4) are metabolites of intestinal microorganisms that act as signaling molecules to strengthen the intestinal barrier and modulate immunomodulatory functions [52].

The Toll-like receptor (TLR) affects the activation of the nuclear factor-κB (NF-κB) pathway, which, together with the secretion of pro-inflammatory cytokines (IL-1β, TNF-α, and IL-6), ultimately leads to the development of a pro-inflammatory response in ionizing radiation-induced epithelial injury [87,88]. SCFAs can inhibit the secretion of the pro-inflammatory cytokine IL-6 by inhibiting the NF-κB signaling pathway, and C4 can stimulate the expression of anti-inflammatory cytokine IL-10 through the homologous pathway GPR109A in IECs, suggesting that SCFAs can effectively alleviate radiation-induced intestinal inflammatory responses [85,89,90].

C4 has been shown to support the integrity of the intestinal barrier, promote the differentiation of colonic regulatory T cells, and exert anti-inflammatory effects [91,92]. C4 promotes gene activation of epithelial integrity-maintaining Glut1 (Slc2a1), Pgk1, and multidrug resistance protein 1 (MDR1), which maintain epithelial integrity, and increases the integrity of small intestinal villi and the number of cup cells, which attenuates RIID by facilitating the assembly of tight junctions and enhancing mucus secretion [93,94,95]. Butyrate and propionate promote anti-inflammatory pathways by inhibiting histone deacetylases (HDACs), resulting in increased histone acetylation and altered gene expression, an epigenetic modulation that is critical for maintaining a balanced immune response and attenuating radiation-induced inflammation [96,97].

#### 4.2.2. Indoles and Their Derivatives

Indole or indole derivatives produced by tryptophan metabolism in *Escherichia coli* and other intestinal microbiota are signaling molecules in the intestinal tract that have a variety of protective effects on intestinal epithelial cells and may limit infrared-induced intestinal inflammation [98,99]. Indole derivatives such as indole-3-carbinol (I3C) and indole-3-propionic acid (IPA) contribute to the maintenance of intestinal homeostasis and can interact with the aryl hydrocarbon receptor (AhR), the activation of which plays a crucial role in modulating immune responses, promoting epithelial barrier function, and preventing inflammatory damage [100].

Indole and its derivatives can enhance the integrity of the intestinal barrier, thereby mitigating RIID [101,102,103,104]. IR damages rapidly dividing intestinal epithelial cells, leading to increased permeability, weakened mucus layer and impaired barrier function. Indole helps to strengthen the tight junctions between epithelial cells, thereby decreasing permeability, preventing harmful substances from entering the bloodstream from the intestinal lumen, and avoiding the exacerbation of inflammatory reactions [101,105]. At the same time, indoles and their derivatives have potent anti-inflammatory properties. They inhibit the expression of pro-inflammatory cytokines and chemokines, thereby alleviating intestinal inflammation. Also, they promote the differentiation of regulatory T cells (Tregs) and the production of anti-inflammatory cytokines, such as IL-10, through the activation of AhR [101,106,107]. This shift to an anti-inflammatory state helps reduce the chronic inflammation associated with radiation enteropathy. Indole promotes the proliferation and differentiation of intestinal stem cells and helps the intestinal mucosa to recover after radiation damage, thus alleviating the depletion of intestinal stem cells caused by IR, which is essential for stem cell renewal and repair of the epithelial lining [108,109].

IPA protects against gastrointestinal toxicity by preserving the intestinal bacterial conformation and small intestinal protein profile of radiation-challenged hosts while activating intestinal PXR/ACBP signaling, preventing radiation-associated hematopoietic syndromes and gastrointestinal syndromes without accelerating tumor growth [102]. Recent studies have shown that I3A enhances the proliferation and differentiation of Lgr5 intestinal stem cells, maintains intestinal barrier integrity, and attenuates mucosal damage. As an important tryptophan metabolite, I3A promotes proliferation of intestinal epithelial cells through the AhR/IL-10/Wnt signaling pathway and up-regulates the abundance of probiotics for the treatment of radiation-induced enteropathy [103]. Meanwhile, I3A effectively ameliorates radiation-induced enteropathy by accelerating the recovery of peripheral blood cells, promoting the recovery of bone marrow cells, and enhancing functional hematopoietic stem and progenitor cells (HSPCs). I3A effectively improves radiation-induced hematopoietic injury by accelerating peripheral blood cell recovery and enhancing functional hematopoietic stem and progenitor cell (HSPC) regeneration. Meanwhile, it can inhibit the production of intracellular reactive oxygen species (ROS) and thus inhibit the apoptosis of HSPCs induced by radiation exposure [110].

#### 4.2.3. Other Intestinal Microbiota Metabolites

In addition to short-chain fatty acids and indoles and their derivatives, a number of intestinal microbial metabolites can likewise mitigate IRRD. The gut flora transforms ellagitanninin in pomegranates, strawberries, and walnuts into urolithin A (UroA), which reduces radiation damage to the gut by decreasing p53 expression, inhibiting caspase 8 and caspase 3 overexpression, and remodeling the gut microbiota to improve the maintenance of gut homeostasis and regeneration. PGF2α activates the FP/MAPK/NF-κB axis to promote cell proliferation and inhibit apoptosis with radiation stress, counteracts infrared-induced alveolar structural damage and collagen accumulation, and reduces post-irradiation pro-inflammatory factors (TGF-β1, IL-1β, and TNF-α), preventing and mitigating IR injury [111]. l-Histidine and its secondary metabolite imidazole propionate (ImP) activate different signaling pathways while reducing the expression of GSDMD, TNF-α and NF-κB increased by irradiation. They also play the function of radioprotection by inhibiting pyroptosis to resist radiation-induced damage [112]. Other metabolites such as tryptophan, quinolinic acid, polyamines, and bile acids can similarly provide protection to tissues by enhancing intestinal epithelial barrier function, modulating immune responses, thus preventing and alleviating RIID.

## 5. Therapeutics Based on Gut Microbiota and Its Metabolites

**Figure 1:**
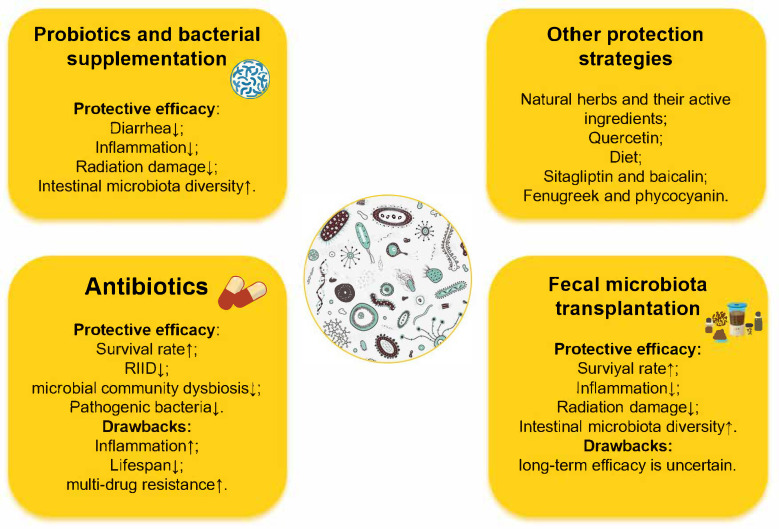
Therapeutics based on gut microbiota and its metabolites intervention.

Because of medical exposures and radiological and nuclear public health emergencies, the development of safer and more effective protection strategies to prevent and treat IR damage to humans has become critical. The following section describes the safety and efficacy of widely used strategies for protection against radiation toxicity: probiotics and bacterial supplements, antibiotics and fecal microbiota transplantation and so on (Figure 1).

### 5.1. Advantages of Gut Microbes and Their Metabolites in Mitigating Radiation Damage

Many technological and biological strategies are currently being developed to prevent and mitigate radiation damage to the gut, such as prophylactic surgical techniques, endoscopic treatments, topical treatments, anti-inflammatory drugs, and biological, chemical, and pharmacological therapies [14]. However, all have certain limitations and drawbacks, such as the possibility of compromising mucosal healing with potential side effects, or leading to microflora dysbiosis or barrier dysfunction, and even high morbidity and mortality associated with treatment options such as surgery [113]. As mentioned earlier in the paper, dysregulation of the ecological niche of the gut flora plays an important role in the severity of radiation-induced gastrointestinal tract damage. While radiation duration, radiation level and type of radiation all affect changes in the gut flora, a symptomatic approach to restoring the optimal microbial composition may be more effective. The intestinal flora and its metabolites have been shown to have long-term radioprotective effects, which are more prominent in maintaining and restoring the integrity of the intestinal barrier, anti-inflammatory properties and restoration of the microbial homeostasis than traditional pharmacological methods. The traditional pharmacological methods are always associated with adverse effects after prolonged use, suggesting that intestinal flora and its metabolites might be beneficial even in the presence of intestinal inflammation, meeting the criteria of an ideal therapeutic agent for radiological protection.

### 5.2. Probiotics and Bacterial Supplementation

Probiotics are a group of active microorganisms that colonize the gut, restore the balance of the gut microbiota, and support overall health by modulating the immune response and maintaining the integrity of the gut, mainly including *Lactobacillus*, *Clostridium casei*, *Bifidobacterium*, *Actinomyces* and *Lactobacillus acidophilus* [114,115,116].

*Lactobacillus* species and their components primarily influence the immune response by facilitating the exchange of immune signals between the gastrointestinal tract and other organs [117]. *Lactobacillus reuteri*, known as a second-generation probiotic, can stabilize the number and capacity of Lgr5 intestinal crypt stem cells and protect intestinal microvascular endothelial cells by releasing IL-22, which directly inhibits the growth of intestinal pathogens. Pepoyan et al. found that *Lactobacillus rhamnosus* Vahe and *Lactobacillus delbrueckii* IAHAHI were the most promising radioprotective probiotics at radiation doses less than 20 Gy, and that they mitigated radiation damage by affecting leukocyte and glucose levels, and significantly reduced the symptoms of radiation-induced diarrhea [118]. *Lactobacillus rhamnosus* GG contribute to the protection and repair of the intestinal mucosa from radiation-induced damage through the release of lipoteichoic acid, macrophage activation and the migration of mesenchymal stem cells, and together with *Bifidobacterium* longum, produces SCFAs and enhances epithelial repair [119,120]. Meanwhile, standard doses of *Lactobacillus acidophilus* LAC-361 and *Bifidobacterium longum* BB-536 reduce grade 2, 3, and 4 diarrhea due to radiation enteritis in surgical patients [121].

A new generation of probiotics, including bacterial strains such as *Akermansia muciniphila* and *Faecalibacterium prausnitzii*, which are naturally occurring in the human gut but have not traditionally been used as probiotics, maintain intestinal barrier function and have anti-inflammatory properties [122,123,124]. Studies in animal models have shown that supplementation with these bacteria reduces intestinal permeability and attenuates inflammation following radiation exposure [125,126].

Combinations of probiotics have been used safely and extensively in the treatment of gastrointestinal disorders. The mixtures of prebiotics (nondigestible food ingredients that selectively stimulate the growth or activity of beneficial gut microorganisms, such as oligosaccharides, inulin, and resistant starches) and probiotics (oligogalactose, *L. acidophilus*, and *Lactobacillus casei*) have shown a positive effect in repairing damage from IR and in protecting patients with radiation-induced diarrhoea, and probiotic complexes have also been shown to play a positive role in acute RIID [37,127].

### 5.3. Antibiotics

Antibiotics, which treat individuals by killing and inhibiting pathogenic bacteria in their bodies, have a major impact on the restoration of the gut microbiota and have long been used to control infections in patients with compromised immune systems [128,129,130]. Some researchers have linked the antimicrobial properties of antibiotics to intestinal bacterial infections following radiation, suggesting that eliminating intestinal microbes with antibiotics may eliminate radiation effects [9,131]. Zhao et al. conducted a study on the specific mechanisms by which antibiotics reduce RIID, and their results showed that both antibiotic cocktail and metronidazole were beneficial in rebalancing the dysbiosis of the gut microbiota after radiation [132,133]. The antibiotic cocktail effectively mitigated radiation-induced microbiota disruption and intestinal damage by inhibiting the TLR4/MyD88/NF-κB signaling pathway, decreasing LPS levels, modulating macrophage polarization, and regulating multiple cytokines [133]. Studies have shown that administration of ciprofloxacin, an antibiotic targeting Gram-negative bacteria, reduces systemic bacterial translocation and improves survival after radiation exposure in mice, suggesting that some antibiotics can selectively target pathogenic bacteria without severely damaging beneficial microorganisms [134,135]. These findings suggest that antibiotic therapy has good results in targeting RIID.

Although antibiotics are essential for the treatment of ionizing radiation-induced dysbiosis and the prevention of bacterial infections, they may also have an indirect effect on the composition of the gut microbiota in different individuals and may instead promote an inflammatory response [136,137,138]. Exposure of wild-type mice to antibiotics and their subsequent monitoring for more than 700 days found that early exposure to antibiotics disrupted the development of the gut microbiota, leading to an increased inflammatory response and a shorter lifespan [139]. Notably, both epidemiologic studies and experimental data suggest that the effects of antibiotics may accumulate in the body. In the long run it may lead to the development of drug resistance. The elimination of commensal bacteria plays a key role in maintaining intestinal integrity, regulating immune responses, and nutrient metabolism. Ultimately, antibiotics can severely alter the structure of the intestinal microbiota, which can adversely affect intellectual performance and health [140,141,142,143]. On the other hand, antibiotic therapy may cause bacterial infections resulting from damage to the intestinal lining, and this damage can impair the intestinal barrier function and allow pathogenic bacteria to invade the bloodstream [144,145].

In conclusion, antibiotics are effective against intestinal bacterial infections caused by abdominal radiation, but they should be applied in a standardized manner in order to avoid the emergence of a resistance crisis.

### 5.4. Fecal Microbiota Transplantation

Fecal microbiota transplantation (FMT) involves extracting functional bacteria from the feces of healthy individuals through procedures such as centrifugal filtration and transplanting them into the gastrointestinal tract of a patient to restore balance to the dysbiotic intestinal microbiota for therapeutic purposes [146,147]. FMT has attracted attention as a potential treatment for a wide range of disorders related to dysbiosis of the intestinal flora [148,149,150]. Generally, FMT is used for the treatment of recurrent *Clostridioides difficile* infections. However, it has also been reported to be effective in ulcerative colitis and irritable bowel syndrome, where it improves the intestinal microbiota and reduces intestinal inflammation, thus potentially mitigating RIID.

Xiao et al. observed higher levels of microbial-derived IPA in the feces of irradiated mice subjected to FMT, which reduced chronic inflammation, lowered the risk of hematopoietic organ damage and bone marrow suppression, and improved gastrointestinal function and epithelial integrity [102]. Wang et al. found FMT to be an effective treatment for chronic radiculitis, and patients experienced significant relief of clinical symptoms after two courses of FMT, which demonstrated the short- and long-term efficacy of the therapy, while the shift from pathogenic to beneficial bacteria with relief of clinical symptoms illustrated the therapeutic potential of FMT. A study showed that FMT increased survival and peripheral white blood cell counts and improved gastrointestinal function and intestinal epithelial integrity in irradiated mice [151]. Meanwhile, FMT preserved intestinal bacterial composition in a sex-specific manner and preserved the mRNA and long-stranded non-coding RNA expression profiles of the host small intestine. Meanwhile, FMT promoted angiogenesis but did not accelerate the proliferation and growth of cancer cells in vivo [151].

All of the above studies suggest that FMT is a promising treatment for modifying microbial communities. However, there are risks associated with the procedure, including the potential spread of infectious agents and the lack of standardized protocols for donor selection, screening and fecal disposal [152,153]. In addition, the long-term effects of FMT are not yet fully understood, and more research is needed to determine its safety and efficacy in different patient populations, including radiation-injured patients.

### 5.5. Other Protection Strategies

The host and diet can control and influence the growth of the gut flora, which has been established as a method of preventing and mitigating IR-induced injury by remodeling IR-induced dysbiosis of the gut bacterial ecology. Natural herbs and their active ingredients can inhibit radiation-induced tissue damage and promote repair. And its mechanism of action is multi-targeted, multi-layered and, most importantly, with minimal side effects [154,155]. It has been shown that baicalin have radioprotective properties and can substantially rebalance radiation-induced flora dysbiosis [156]. Quercetin may mitigate IR injury by modulating the gut microbiota and reducing inflammatory cytokine levels [157]. Strategies such as sanguinarine and phycocyanin can reduce inflammatory cytokine levels by reducing LPS-producing bacteria or blocking the TLR4/Myd88/NF-B pathway [158].

These therapies provide good concepts for protection and repair of radiation damage (Table 3). However, their safety and the stability of potential therapeutic effects are uncertain and therefore need to be further explored in future studies.

## 6. Conclusions and Future Directions

RIID is a complex and challenging condition that has a significant impact on patients undergoing radiotherapy. Understanding the mechanisms, risk factors, and clinical manifestations of RIID is essential for developing effective management and prevention strategies. IR causes radiation enteritis by disrupting the immune system, disrupting the balance of the intestinal microbiota, and damaging the intestinal and vascular endothelium. The gut flora plays a crucial role in the etiology and treatment of radiation-induced injuries. As irradiation promotes the proliferation of harmful gut microbes, increasing the risk of intestinal diseases, beneficial bacteria that naturally inhabit the gut can directly affect the intestinal epithelium or modulate the intestinal immune system, thereby providing radiation protection. This review emphasizes the key role and mechanisms of the gut microbiota and its metabolites, as well as the complex interactions between the microbiota and radiation potency and injury, providing promising therapeutic avenues—microbiota-based therapies such as probiotics, FMT, antibiotics, and others—which have been discussed as a potential strategy for controlling radiation-induced injury. However, at the same time, RIID can be further exacerbated by reductions in probiotic populations and expression of their metabolites, etc. Despite the challenges, the potential benefits of microbiota-based therapies are substantial. Future research should focus on elucidating the specific mechanisms by which the gut microbiota influences radiation injury, optimizing therapeutic strategies. By deepening our understanding of the gut microbiota and its metabolites, we can develop innovative therapeutic approaches to prevent and manage radiation-induced injury, thereby improving patient outcomes and quality of life.

## Figures and Tables

**Table 1:** Radiosensitivity of representative gut-associated bacteria to ionizing radiation.

Bacterial Species	Radiation Type & Energy	Sensitivity Index (D10 kGy)	References
*E. coli*	Low-energy X-ray, 70 kV	0.040–0.078	[74]
	γ	0.24–0.35	[75]
*Bacillus* spp.	e-beam/γ	0.31–0.37	[76]
	Low-energy X-ray	3.3	[77]
*Lactobacillus* spp.	IR at clinical RT doses	relatively resistant	[78]
	^60Co γ	0.526	[79]
*Clostridium perfringens*	γ (Direct X-ray D10 data limited)	0.40–0.80	[80]

**Table 2:** Changes in the gut microbiota and its metabolites after radiotherapy.

Radiation Type	Radiation Level	Radiation Time	Objects	Biospecimen	Analytical Techniques	Changes in the Gut Microbiota and Its Metabolites	References
total body radiation	8.2–9.2 Gy	290 days	C57BL/6 mice	Feces	16 S rRNA gene sequencing	*Lachnospiraceae* ↑, *Enterococcaceae* ↑; short-chain fatty acids (acetate, butyrate, and propionate) ↑, I3A and KYNA ↑	[82]
X-ray	5, 12 Gy	30 days	C57BL/6J mice	Feces	16 S rRNA gene sequencing	*Lactobacillaceae* ↑, *Staphylococcaceae* ↑ *Lachnospiraceae* ↓, *Ruminococcaceae* ↓, *Clostridiaceae* ↓; glyceric acid ↓, homogentisic acid ↓, glutaconic acid ↓ and pipecolic acid ↓ hippuric acid ↑, taurine ↑, urobilinogen ↑	[83]
pelvic radiotherapy	1.8–2.0 Gy/day	five times a week during a 5 week period	11 cancer patients	Blood	16 S rRNA gene sequencing	* Firmicutes * ↑, *Bacteroidetes* ↓	[72]
60Co γ-ray	8 Gy	single	C57BL/6 mice	The small and large intestinal contents	16 S rRNA gene sequencing	*Alistipes* ↑, *Lactobacillus* ↑, *Akkermansia* ↑ *Barnesiella* ↓, *Prevotella* ↓, *Bacteroides* ↓, *Oscillibacter* ↓, *Pseudoflavonifractor* ↓, *Mucispirillum* ↓	[12]
X-ray	18 Gy	Single	C57BL/6 J mice	Intestinal biopsy feces	16 S rDNA sequencing UHPLC-MS	* Bacteroidetes * ↓, *Firmicutes* ↓; Significant changes in CerG1, CerG3GNac1, cPA, DG, dMePE, FA, ganglioside GM3, LdMePE, LPC, LPE, LPG, LPI, PC, PE, PG, PI, SM, So and the acute phase is the opposite of the chronic phase	[83]
60Co γ-ray	4 Gy (non-myeloablative)/8 Gy (myeloablative)	Single	BALB/c mice	Cecal contents	16 S rRNA gene sequencing	* Bacteroidaceae * ↑ *Ruminococcaceae* ↓, *Lachnospiraceae* ↓, *Lactobacillaceae* ↓, *Defluviitaleaceae* ↓, *Peptococcaceae* ↓, *Christensenellaceae* ↓ acetic acid ↓, valeric acid ↓	[84]
223Ra, 99mTc			2 healthy volunteers	Feces	16 S rRNA gene sequencing	* Firmicutes * ↑, *Proteobacteria* ↑ *Bacteroidetes* ↓, *Actinobacteria* ↓	[85]

↑ Increase; ↓ Decrease.

**Table 3:** Microbiota-based and metabolite-targeted therapeutics for RIID.

Intervention Type	Category	Mechanism of Action	Efficacy and Outcomes
Probiotics and Bacterial Supplementation Therapy	Traditional Probiotics	Regulate immune signal transduction Stabilize crypt stem cells to protect intestinal microvascular endothelial cells Modulates leukocyte counts and blood glucose levels Activates macrophages, and promotes mesenchymal stem cell migration	Significantly reduces radiation-associated diarrhea incidence, promotes intestinal mucosal repair and regeneration
	Next-Generation Probiotics	Maintains intestinal barrier integrity and exerts anti-inflammatory activity	Reduce intestinal permeability and alleviate inflammatory responses
	Probiotic-Prebiotic Complex Formulations	Repair tissue damage and selectively promote the proliferation and metabolic activity of beneficial gut microbiota	Effectively repairs IR-induced injury, protects patients with radiation-induced diarrhea, and demonstrates clear improvement in acute RIID
Antibiotic Therapy	Broad-spectrum and Targeted Antibiotics	Reduces LPS levels Modulates macrophage polarization and cytokine networks Improves intestinal dysbiosis	Effectively control intestinal bacterial infections, alleviate dysbiosis and intestinal tissue damage
FMT	Fecal Microbiota Transplantation Technology	Restore gut microbiota balance Increase IPA production and promote angiogenesis Regulate host small intestine mRNA and long non-coding RNA expression profiles	Reduces chronic inflammatory responses and hematopoietic organ damage risk, improves gastrointestinal function and epithelial integrity Alleviates clinical symptoms of chronic radiation enteritis
Other Protective Strategies	Natural Herbal Active Components	Rebalances radiation-induced dysbiosis Reduces inflammatory cytokine levels Inhibits inflammatory cascade reactions	Exhibits definite radioprotective activity and mitigates IR-induced tissue damage

## Data Availability

No new data were created or analyzed in this study.
